# Deep learning mitigates batch effects in Raman spectroscopy

**DOI:** 10.1038/s41598-026-62086-1

**Published:** 2026-10-26

**Authors:** Joel Sjöberg, Adrian Lita, Mioara Larion, Ion Petre

**Affiliations:** 1 Department of Mathematics and Statistics, University of Turku, Turku, Finland; 2 Neuro-Oncology Branch, National Cancer Institute, National Institutes of Health, Bethesda, 20892 USA

**Keywords:** Batch effect, Deep learning, Feature importance, Glioma, Raman spectroscopy, Cancer, Computational biology and bioinformatics, Mathematics and computing

## Abstract

Raman spectroscopy is widely used for its ability to provide rich molecular-level information from biological samples. However, the high sensitivity of Raman spectra can also unintentionally encode extraneous information, such as patient identity, instrument-specific signatures, environmental conditions, and sample handling protocols. This hidden structure, referred to as the batch effect, poses increasing challenges in machine learning applications, where it can lead to model overfitting, poor generalization, and even privacy risks when datasets are shared. In this work, we investigate the presence and impact of the batch effect in a Raman spectroscopy dataset derived from glioma tumor samples. We show that deep learning can accurately predict patient identity from individual spectra, revealing strong bias patterns in the data. To mitigate this issue, we explore several approaches and introduce an algorithmic framework based on adversarial deep learning. Our method effectively reduces the influence of confounding sample-specific signals while preserving information relevant to tumor classification. These findings highlight the need for careful bias control in Raman-based AI pipelines and provide tools to support more robust applications in biomedical spectroscopy.

## Introduction

Utilizing machine learning (ML) to perform highly advanced analyzes and to aid experts in formalizing patient diagnoses continues to be one of the many promising aspects of utilizing artificial intelligence (AI) within the medical field. This frontier continues to expand at a rapid pace, in no small part due to the expansion of openly available datasets through resources such as The Cancer Genome Atlas (TCGA) and so-called Kaggle competitions. Due to the maintained interest in deep learning, many different avenues have opened up for highly complex deep learning models applied to large datasets of various cohorts^1–3^. The increase in available datasets has led researchers to develop models on combinations of these datasets. However, recent studies have shown how models trained on combinations of different datasets develop a type of bias, allowing the models to learn patterns correlated with the original experimental designs rather than the research-specific patterns they were intended for^4–9^. This is often referred to as the *batch effect*, a form of sample-specific bias.

The batch effect is not caused by a lack of proper acquisition strategies or sample preparation protocols. Rather, the issue is caused by the high sensitivity of the instruments, particularly Raman spectrometers, which may include systematic, non-biological differences that are not trivially noticeable. This may be caused by the specifics of the instrument and its setup, by the experimental conditions (including day-to-day drift in Raman measurements, laser power fluctuations, and light intensity in the experimental room), by sample preparation protocols (thickness of the sample, cell culture or sample preservation conditions, and storage medium), or by subtle changes between operators or labs (in Raman experiments, variation in focusing, calibration, denoising, and baseline correction). Our investigation shows that this effect is not exclusive to data from different acquisition sites; it is also reflected in Raman data from the same lab, extracted at different times. We view the batch effect as yet another artifact, similar to noise, baseline signals, and cosmic ray spikes in Raman data, that must be acknowledged, rectified, and accounted for in the computational design.

Raman spectroscopy is a non-destructive analytical technique used to probe molecular vibrations within materials, producing spectra that serve as distinctive molecular fingerprints of the sample. The high informational content of Raman spectra aligns naturally with modern computational approaches, including statistical analysis, machine learning, and deep learning methods^10–12^. In recent years, the integration of Raman spectroscopy with artificial intelligence has enabled a range of impactful applications in oncology. These include label-free molecular profiling^13^, intra-operative tumor margin assessment^14,15^, tumor classification and molecular sub-typing^16–18^, and monitoring of treatment response^19–21^.

**Fig. 1:**
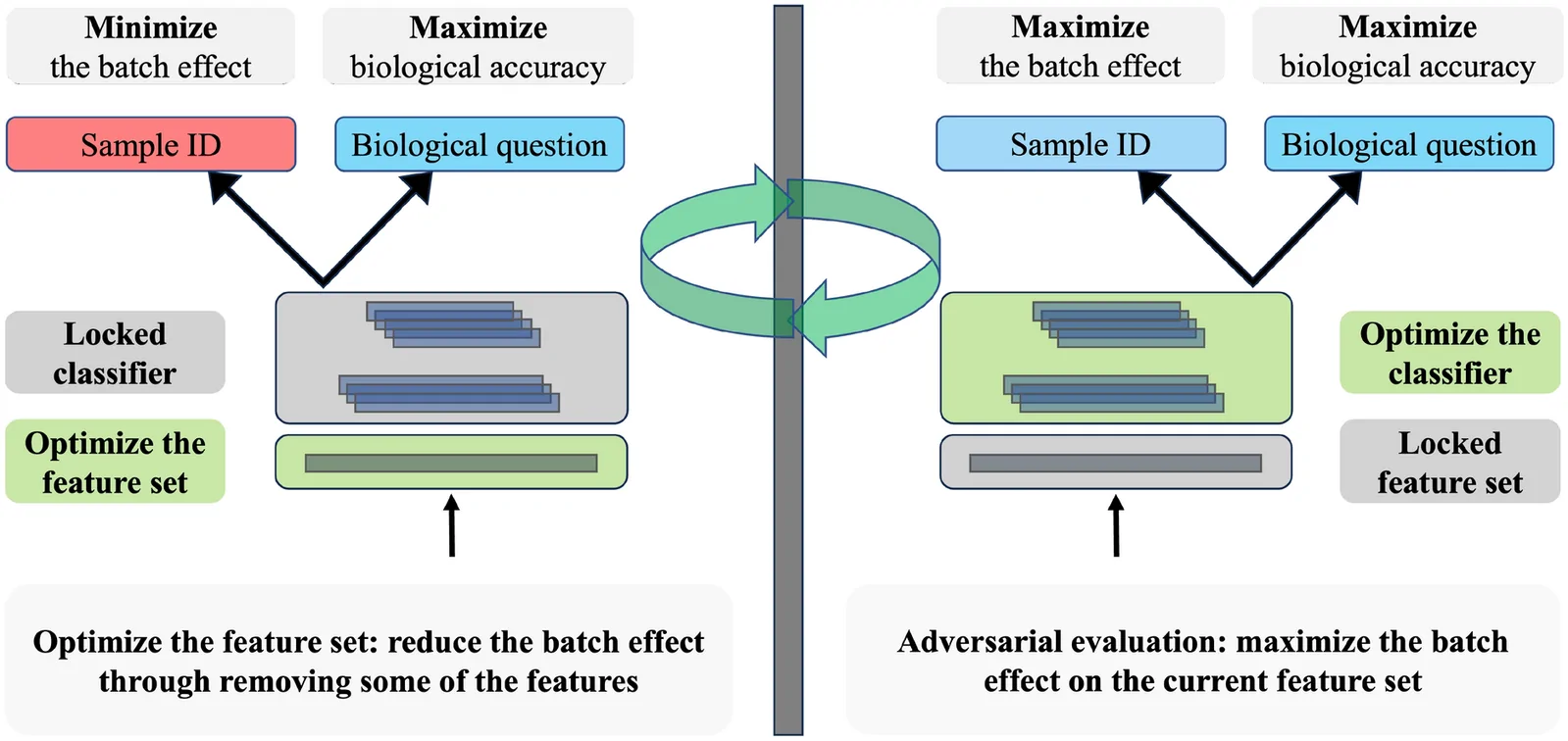
Our computational pipeline to reduce the batch effect on Raman spectroscopy data. We adopt an adversarial learning approach based on two model components. The first one is a feature importance layer, whose objective is to optimize the feature set to be processed by the rest of the model. The second component is a classifier working on the optimized feature set to maximize the accuracy of the biological question under study. The model is trained in an adversarial fashion. With the classifier locked, we train the feature importance layer to minimize the batch effect, while maximizing the biological accuracy. The adversarial step locks the feature importance layer and it evaluates its batch effect by training the classifier to maximize both the batch effect, and the biological accuracy. The two update steps are iterated until convergence. Pseudocode describing this process along with the preprocessing methods are available in the Supplementary material, Algorithm 1.

In this study, we investigate the batch effect in Raman spectra acquired from glioma tumors. Our analysis is based on the APOLLO dataset^16^, which contains Raman measurements from 59 glioma samples representing various tumor subtypes collected from 46 unique patient-sample ids. We demonstrate that Raman spectroscopy data is so rich and sensitive that a machine learning model can accurately predict patient identity solely from the spectral signatures. This highlights a significant batch effect in the data.

To address this, we evaluate several strategies aimed at reducing this bias while preserving the ability to accurately classify the tumor type. Specifically, we assess whether the predictability of patient identity decreases after debiasing without compromising performance in identifying the glioma subtype. Our approaches include classical spectral correction techniques (such as baseline correction, cosmic ray removal, and denoising) as well as deep learning-based methods. In the latter, we design encoder-based and binary-headed deep neural network models trained in an adversarial fashion (Fig. 1). The encoder is optimized to retain features predictive of IDH mutation status while suppressing those informative for patient identity, thereby eliminating sample-specific patterns responsible for identity recognition.

We quantitatively evaluate the effectiveness of these methods in reducing bias while maintaining clinically relevant classification performance. We provide an example of how to approach batch effect reduction in Raman spectroscopy datasets. This can be done by first identifying classification tasks that prove the presence of biased data patterns in the project. Then, the biased labels are utilized to train a model containing our feature importance layer to dynamically curate the input signal. Our results show that this can reduce the bias within feature-rich Raman spectroscopy data of glioma tumors. Our contribution to this ongoing effort is in the strategy for leveraging adversarial training to discover relevant features with respect to biological classification while removing features relevant for biased patterns responsible for the batch effect.

## Related work

The cause of the batch effect originates from heterogeneous data distributions, which result from differing experimental setups^22,23^. This has raised concerns regarding the use of deep learning methods applied to larger datasets^7,24,25^, and the topic continues to be studied in various contexts using statistical methods^26^ and machine learning methods^27–29^. Research focused on removing the batch effect has expanded in the context of RNA-sequencing data. The review in^30^ applied six standard methods for batch effect mitigation on two simulated datasets, separated by the severity of the batch effect. Their results showed that the so called ComBat method^23^, utilizing the empirical Bayes algorithm, outperformed other methods in reducing the batch effect. The BBKNN method^31^ has seen successful applications in reducing the batch effect for sequence data by forming graphs in the principal component analysis reduced space among multiple batches of data. To better track the mitigation process performed by these models, the BEER method^32^ was introduced with the aim of providing explanations for how the batch effect was reduced while improving the performance of BBKNN.

The use of deep learning methods for addressing the batch effect in data is a more recent direction. Kheiri et al.^33^ showed that whole slide images of two different cancers, taken from two differing acquisition sites, could be used to train a neural network model for predicting cancer type and acquisition site in tandem. They proved that the color staining, used differently at each acquisition site, partially caused the batch effect and retrained their model using gray-scale images with added noise. The accuracy of acquisition sites diminished after processing the data, while cancer type accuracy was maintained, showcasing a reduced impact of the batch effect in their dataset. Their results indicate the need for intuitive deduction in identifying possible causes of the batch effect. The recent work by Fung et al.^34^ utilized the LassoNet^35^ deep learning model to reduce the batch effect in a microbes dataset. Their approach builds on the observation of Kheiri et al. that the batch effect can be quantified through learning biased data patterns. Their results showed a promising avenue for improving classification accuracy on omics data while reducing batch effect patterns through an autoencoder architecture. Their work measured the batch effect occurring by combining the data from 5 different studies.

## Methodology

### Data

We used the dataset from^16^, consisting of Raman spectra extracted from 46 unique patient tumors. All the samples were provided by Dr. Houtan Noushmehr. All research was carried out in accordance with relevant guidelines and regulations. Patients provided written informed consent to have their specimens used for research purposes. Ethics approvals for this de-identified sample analysis were obtained by relevant institutional and/or licensing committees (Henry Ford Hospital Systems Institutional Review Board #10963, University Hospitals Institutional Review Board #CC296 (case 1307)).

To measure the batch effect through various preprocessing methods, we explored three versions of the data: the raw data; the data after baseline correction, cosmic ray removal, and denoising performed by our recent RADAR architecture model^36^; a manually processed variant identifying the locations of the peaks via the mean spectrum, with the baseline influence of each peak region removed by interpolating a line from the left to the right end of the peak and subtracting it from the raw peak intensities. All spectra in the three datasets were min-max normalized using their individual minimum and maximum values; e.g., a spectrum *s* is normalized by $\frac{s - min(s)}{max(s) - min(s)}$. The distributions of the datasets are shown in supplementary Figure 9 a. Since our tests showed that models had minimal sample ID accuracy when trained on the manually processed data, we decided to utilize it for training the feature importance layer.

Sporadic spectral artifacts were reduced by optimizing the acquisition time. When present, affected spectra were identified during preprocessing and excluded from further analysis. These artifacts were typically associated with laser-induced overheating, tissue damage (e.g., cracks), or the presence of highly fluorescent components such as heme or dried blood. Such regions are described in supplementary Figure 10. We removed any spectrum with intensities outside the truncated wide quantile range of [0.005, 0.995]. This was done by computing the quantile range for every spectral wavelength in the global dataset consisting of all available sample spectra. The spectra whose intensity at any wavelength lay outside the range were marked for removal.Of 300,506 spectra, 39,584 spectra were identified as outliers and removed by the method, leaving 260,922 spectra for the learning tasks. We used the manually preprocessed dataset to locate the positions of outliers in each sample and then removed them from the manually curated, the RADAR preprocessed, and the raw datasets. The distributions of the outliers are shown in Figure supplementary 9 b.

In this project, we aimed to reduce the batch effect occurring from unique patient patterns in our dataset. This required that every patient sample be present in the dataset to enable the learning of all unique patient patterns. For this reason, leave-one-patient-out cross-validation was not used in this project. Instead, we used stratified 10-fold cross-validation. These folds separate each patient sample into a distinct training and test set such that the spectra in the validation set have the same proportion with respect to sample ID. This ensures that each sample is present in the validation set to learn and validate on all patient IDs. In contrast, the IDH1 labels present within our dataset are evenly divided across the samples. This balance is maintained in each fold by focusing on the sample IDs. A look into the diversity of the sample maps is shown in supplementary Figure 10 a, where a principal component analysis (PCA) model is trained for the entire dataset to reduce the data into a 3-dimensional representation. The surface map is displayed as an image by using the 3 dimensions as color values of red, green, and blue. The diversity in the tumor surface can properly be examined after removing the outliers in the samples (supplementary Figure 10 b).

### Isolated feature importance

To assess the contribution of individual spectral features to both sample ID and IDH1 mutation status prediction, we conducted a feature-wise analysis using the manually preprocessed data. Each Raman wavelength was evaluated independently by training two separate neural networks: one for classifying the 46 sample IDs and another for binary classification of IDH1 mutation status (mutant vs. wild-type). The question we explored was which wavelengths are most discriminative of the sample ID and of the IDH1 mutation status simultaneously. We employed decision tree models to solve the classification problems, and balanced accuracy was used to quantify their predictive performances (Supplementary Figure 11).

Certain spectral intensities demonstrated some predictive power when used in isolation to train a decision tree. For example, some individual features achieved over 7.1% balanced accuracy for sample ID classification (higher than the random guess baseline). Similarly, for IDH1 status, several features yielded over 55% balanced accuracy (higher than the random guess baseline). Our conclusion from this experiment was that singular features could not properly capture either ID or IDH1 characteristics to sufficiently predict them. Instead, multiple features are required to adequately model the target values.

### The batch effect in Raman data

**Fig. 2:**
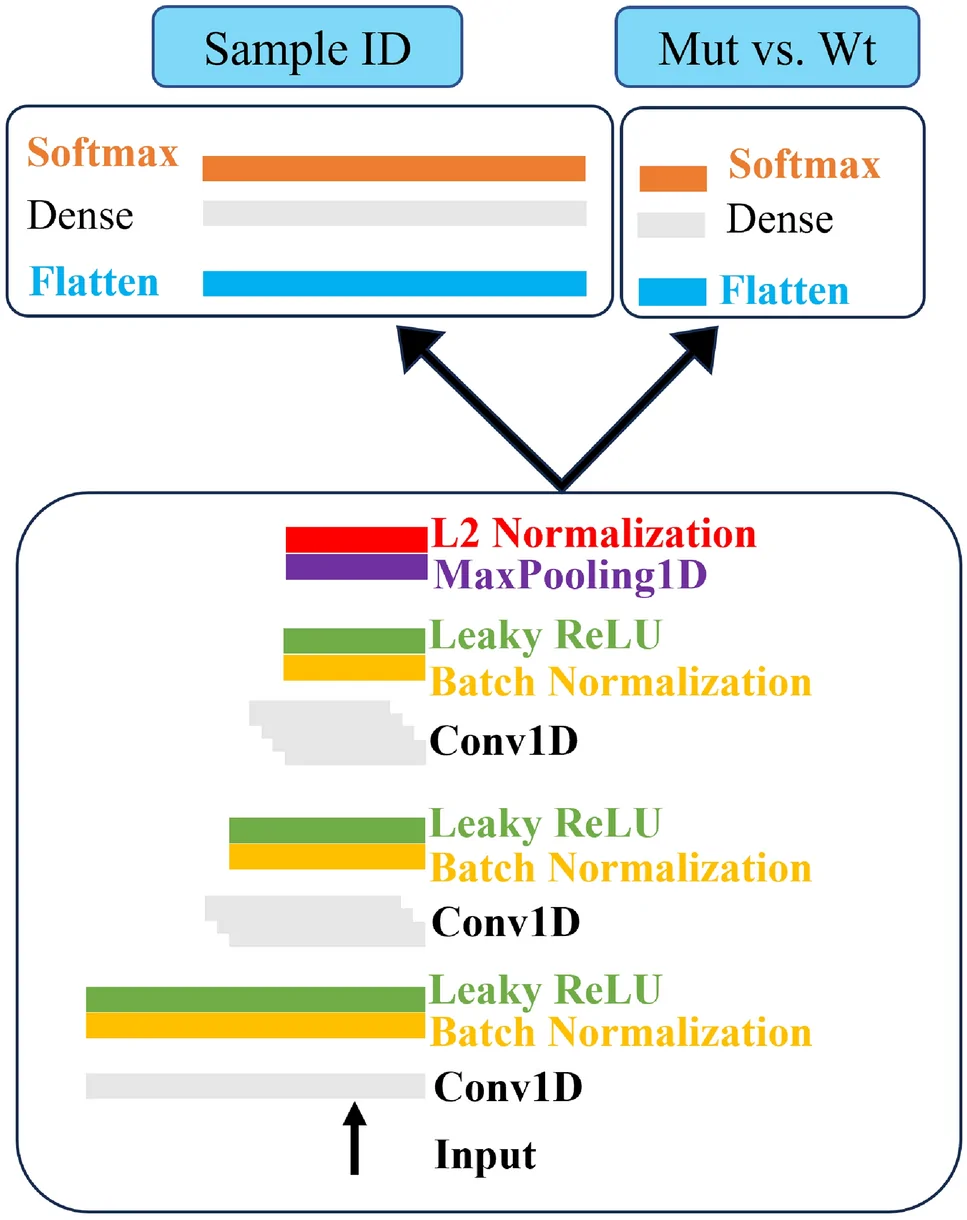
The model architecture used to predict sample ID and mutant vs. wild-type in tandem for the training set. The model is used to detect the batch effect in Raman data, and to reduce the effect through adversarial training. The parameters of each layer are listed in Supplementary Table 1.

**Fig. 3:**
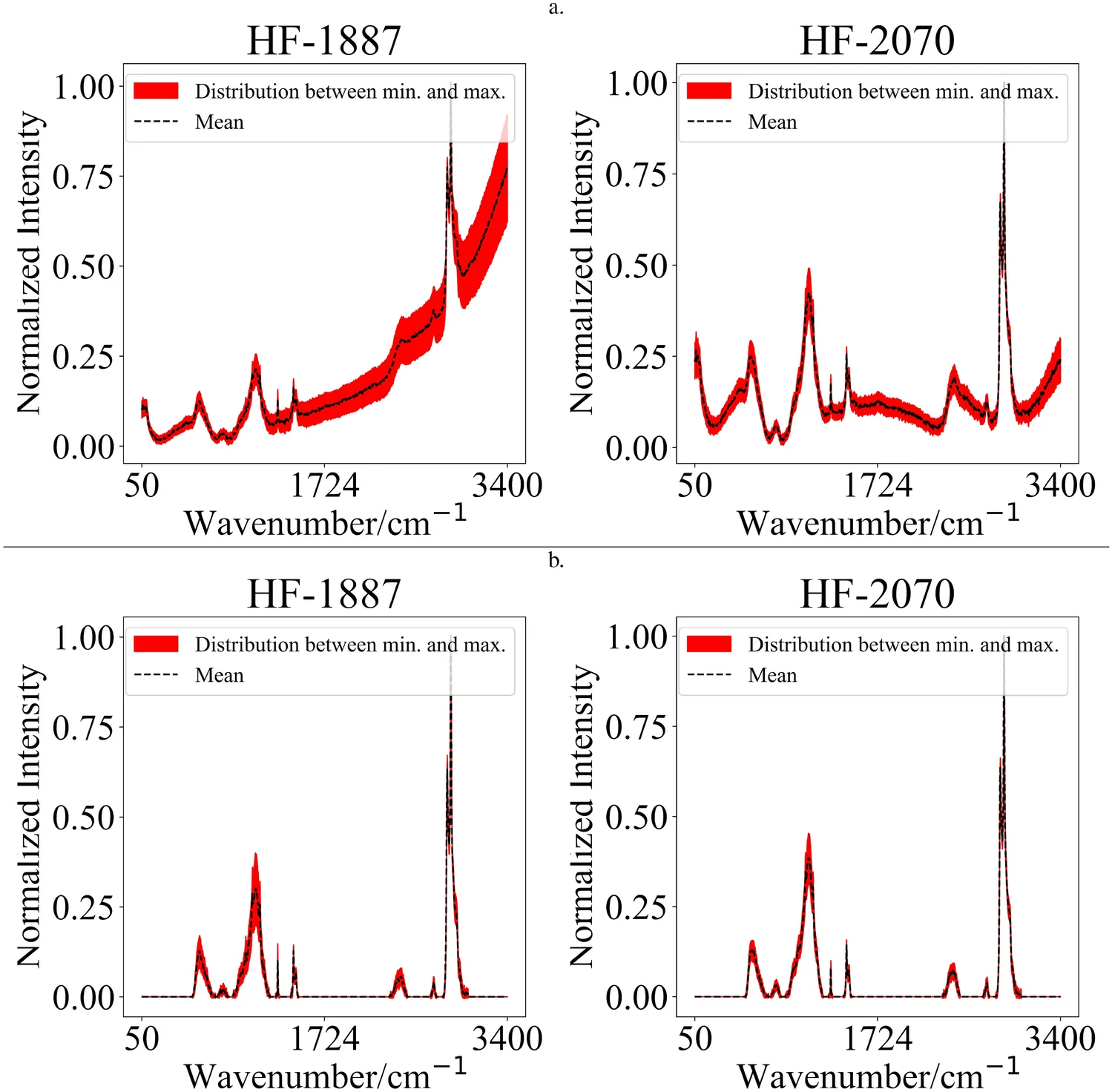
Assessing the batch effect in the APOLLO dataset (^16^): whether Raman spectra could be used to identify their sample of origin. The mean spectra of the two samples are shown in black and the standard deviations from the means are shown in red. In a. is shown the raw spectra using only min-max normalization before training the model. In b. are the same spectra, corrected with manual baseline correction before min-max normalization.

We assessed the batch effect in the APOLLO dataset (^16^) by testing whether individual Raman spectra could be used to identify their sample of origin. To simultaneously evaluate the batch effect and address the biological classification task, we trained a two-headed neural network (Fig. 2) to predict simultaneously the sample ID and the IDH1 mutation status (mutant vs. wild-type) from individual spectra. The summary of the model layers is available in the Supplementary Table 1. As an initial demonstration, we considered a simplified case involving two tumor samples: HF-1887 and HF-2070. Despite sharing the same glioma methylation subtype (IDH1 Mutant), their Raman spectra differ markedly (Fig. 3 a.). The dataset included 4622 spectra from HF-1887 and 2684 spectra from HF-2070, each consisting of 1738 spectral wavelengths.

**Fig. 4:**
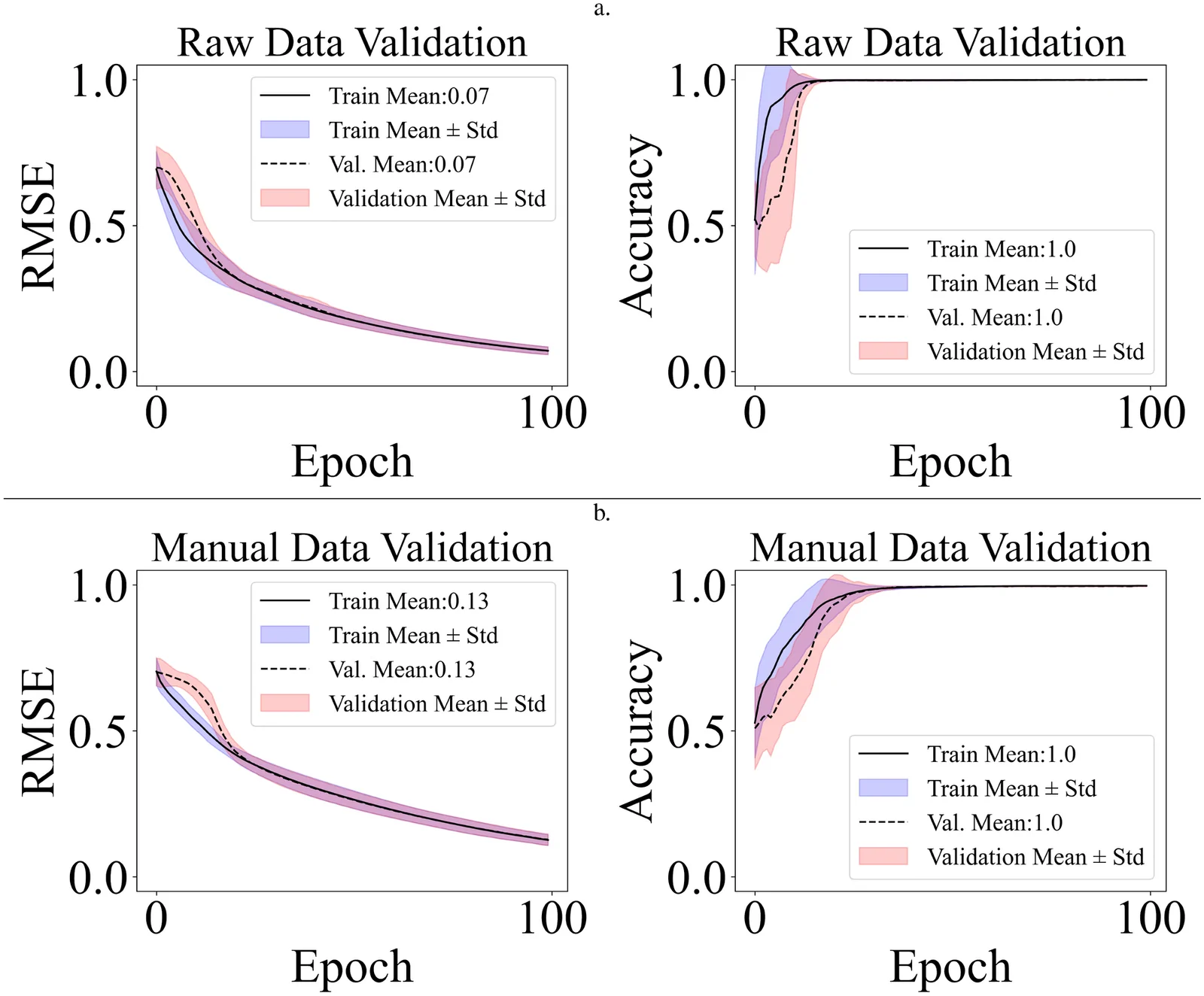
The loss curve and the accuracy histories of the models (light blue: standard deviation of training loss across 10 folds during training, light red: standard deviation of validation loss across 10 folds.) show that the model can easily distinguish between the two samples (AUC = 1.0). regardless of using the raw data (**a.**) or the baseline corrected data (**b.**).

We trained a deep learning model with just 665 parameters to classify the sample ID based solely on the spectral input for samples HF-2070 and HF-1887. The initial experiment showed that the model was able to efficiently find patterns which discriminated between the two datasets in Fig. 3 a. (The training accuracy and loss are displayed in Fig. 4 a.)

We used smaller models to ensure the batch effect was not the result of model overfitting due to the reduced dataset size. The summary describing the model architecture and hyper-parameters are present in Supplementary Table 2. The model successfully distinguished between the two samples (Fig. 4), clearly demonstrating the presence of the batch effect, i.e., the presence of sample-specific patterns that persist even in biologically similar tumors.

We extended our analysis to the full APOLLO Raman dataset consisting of 300,506 spectra collected from 46 patients. For training we used the larger model architecture summarized in Supplementary Table 1 visualized in Fig. 2 The model achieved 68% accuracy in predicting the sample ID (a remarkable level for a 46-class classifier) and 84% accuracy in predicting the IDH1 methylation sub-type (a binary classifier). These results confirm the strong presence of the batch effect in the APOLLO dataset, as the model could reliably identify the sample of origin despite focusing only on single-spectrum inputs.

### Reducing the batch effect via preprocessing

To investigate the impact of data preprocessing on the batch effect, we returned to the simplified setup using samples HF-1887 and HF-2070. We hypothesized that the baseline signal, commonly present in raw Raman spectra, could be a key contributor to the batch effect observed between these two samples. To test this, we applied manual preprocessing to isolate the zero-centred peak regions, effectively removing the baseline components (Fig. 3 b.). Using this baseline-corrected data, we retrained the same model architecture. As shown in Fig. 4 b, the model required more training iterations to learn the sample ID, suggesting a reduction in superficial differences. Nonetheless, it ultimately achieved a high discriminative performance, with an AUC of 1.0, indicating that even after baseline correction, sufficient sample-specific features remained to enable near-perfect classification.

**Fig. 5:**
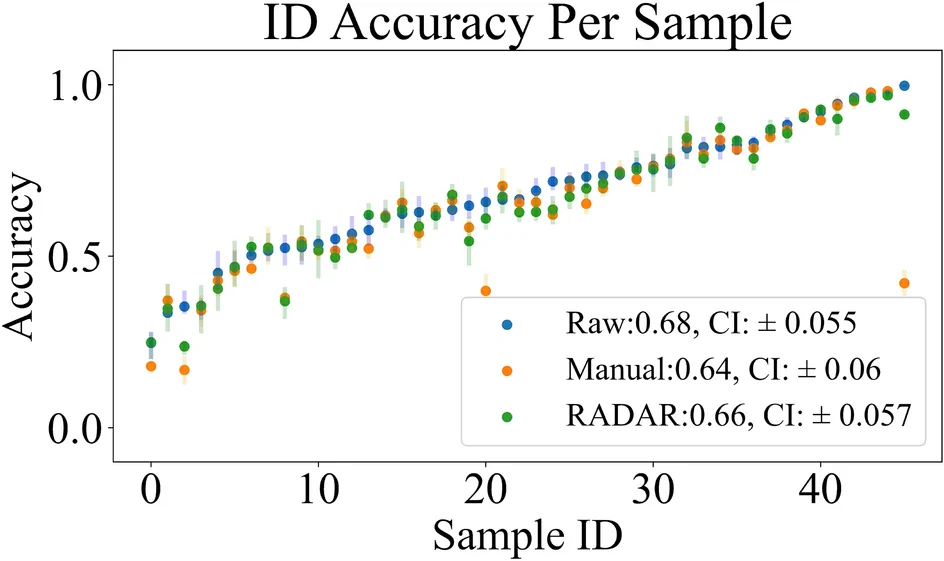
The per-sample ID accuracy across the 10 cross-validation folds, comparing model performance when trained on the raw data versus two preprocessed versions of the data. The vertical lines show the accuracy spread across the 10 validation folds within the inter-quartile range (25, 75).

We next extended our analysis to the full APOLLO dataset, performing multiclass classification to predict the sample ID for each of the 46 patient-derived tumor samples. To evaluate the effect of preprocessing on the batch effect, we applied two preprocessing strategies: our recently proposed RADAR deep learning pipeline^36^, and the same manual baseline correction approach utilized in the simplified setup. For both the raw and preprocessed datasets, we retrained the same two-headed model (Fig. 2) from scratch for 100 epochs, using stratified 10-fold cross-validation. In each fold, the sample weights were computed to simulate a balanced dataset with $\sqrt{\frac{\max _{c}|N_c|}{|N_x|}}$, where $|N_i|$ is the number of spectra belonging to class *i*. We evaluated our approach based on the average balanced accuracy across all folds on their respective validation sets. The models were tasked with predicting both the sample ID and the IDH1 mutation status from individual spectra. As illustrated in Fig. 5, preprocessing generally made it more difficult for the models to learn sample-specific features: the AUC for sample ID prediction decreased from 75% on the raw data to 69% with RADAR preprocessing and to 66% with manual preprocessing. These numbers, however, remain high, showing the continued presence of the batch effect in the preprocessed data. A detailed summary of training and validation accuracies for sample ID and IDH1 predictions across all samples is provided in Supplementary Tables 3, 4, and 5. Supplementary Table 3 contains the per-sample accuracies on the data from the dataset consisting of raw Raman spectra. The per-sample accuracies of the manually processed and the RADAR processed datasets are listed in Supplementary Tables 4 and 5 respectively. The metrics listed are computed as the mean of the respective metric across the 10 validation folds.

### Learnable feature importance

We implemented a novel feature importance layer using *Keras*^37^. Each learnable parameter *w* was initialized as a vector with elements of ones. By using uniform initialization, the baseline assumption is that each feature is equally important when training starts. During training, each element in the feature importance vector *W* is constrained between 0 and 1 i.e. the feature importance element $w_i \in W$ is constrained according to:

$$\begin{aligned} w_i = \frac{|w_i|}{max(1, max(|W|)) + \varepsilon }. \end{aligned}$$

Thus, each *w* is constrained to a real-valued interval between 0 and 1, indicating model reliance on said input feature. This ensures that the parameters stay positive and avoids parameter explosion by forcing a normalized form when gradient descent otherwise would increase the parameters past 1. By constraining each parameter individually, it is also possible for the layer to identify all features as irrelevant by setting them all to 0, which is why $\varepsilon = 1 \times 10^{-7}$ is used to stabilize the division. Furthermore, we implemented a normalization factor *m* used to scale the activations of the layer. This is necessary as $W_n$ can decrease in intensity significantly, leading to unstable learning as the data distribution is scaled down. The parameter *m* is initialized as 1 but is iteratively modified according to the activations, $m = \alpha m + max(z) \cdot (1 - \alpha )$, where *z* is the input vector scaled by the feature importance’s ($X \cdot W_n$) and *α* is a counter initialized at 0. The *α*-counter is incremented by *ɛ* at every update step until it reaches 1, at which point it is assumed that the data distribution has become stable.

The interpolation coefficient *α* is constrained to the interval [0,1]. It is initialized to 0 and increased linearly by 1/#epochs at each training epoch until reaching 1 at the start of the final epoch. Consequently, the scaling factor m evolves smoothly from the initial maximum activation to the stable learned maximum of the feature-importance vector, avoiding abrupt changes in the normalization and promoting stable optimization. The proper output of the layer is then $\frac{z}{m}$. The value of *ɛ* in this work was set to *1e-7* which roughly equates to the inverse number of updates performed during model training. We recommend setting it to the inverse number of updates carried out in novel experiments. To promote feature scaling, the layer weights are subject to $L_1$-regularization with a regularization parameter *λ = 1e-3*. We found that the layer managed to properly reduce the data to important features exclusively for our dataset. We tested the stability of this solution by re-training using *λ = 1e-2* and *λ = 1e-4*. For larger *λ* values, the layer caused diminishing signals in all spectrum features whereas smaller *λ* values decreased at slower rates, requiring longer training times as a result. Therefore, we concluded that *λ = 1e-3* is optimal for our dataset.

We note that a larger parameter may be necessary for data with less than 100 features. We also recommend min-max normalization or $L_1$-normalization as a preprocessing method for this layer to ensure stable performance.

Since feature importance is learned in a task-dependent manner, spectral regions are not suppressed solely because they correspond to high wavenumbers or exhibit lower intensities. Nevertheless, for applications where diagnostically relevant information is concentrated in weaker spectral features, the learned importance distribution may differ. Evaluating the proposed approach on additional datasets and alternative diagnostic tasks will therefore be important to assess its generalizability. In the present dataset, the learned feature importance was concentrated in the fingerprint region, suggesting that the network found this region most informative for the specific IDH1 classification task under the adversarial objective. This should not be interpreted as evidence that the high-wavenumber region lacks diagnostic value in general.

### Adversarial feature learning

Our approach aims to suppress features contributing to sample-specific biases while preserving those that are informative for the biological classification task. For this, we initialize a feature encoder model designed to scale the input features according to their learned importance and a classification model with two output heads. The feature encoder consists of our feature importance layer followed by an average pooling layer, meant to reduce sporadic noise resulting from feature-wise transformations. Our strategy is to first encode the input spectrum using the feature encoder and then feed the transformed version into the classification model. We implemented an adversarial training loop with two alternating update steps to train the two parts (displayed in Fig. 1):

1. **Model update:** The feature encoder model is locked, and the rest of the network is trained to improve performance on both sample ID and IDH1 classification tasks. This step is vital for allowing the model to learn the feature transformed data. At initialization, all feature values are assigned a value of 1, i.e., the feature importance layer is equivalent to the identity function before it is optimized.
2. **Feature importance update:** The feature importance layer is unlocked while the rest of the model is locked. During this step, the feature importance layer is updated to minimize the sample ID signal (using a *negative* categorical cross-entropy loss) and simultaneously enhance IDH1 classification (using a standard *positive* categorical cross-entropy loss).

This adversarial process encourages the model to discard feature patterns associated with sample identity while retaining those relevant to the IDH1 mutation status. After each iteration made out of one training epoch for training both the learnable feature layer and the main model, the learning rate is reduced by 0.3% to reach convergence. The resulting feature encoder model can then be used to transform the original spectra into a de-biased representation, which we feed into the final classification architecture. A description of the pipeline described in this section is available as pseudocode in the Supplementary Material, Algorithm 1.

We trained models using stratified cross-validation in 10 folds on a computer with 32GB RAM and an Nvidia RTX 3050 GPU. using the CPU for data loading. Model training employed the default TensorFlow/Keras data-loading mechanism without explicitly configuring parallel CPU workers. Since the dataset was loaded into memory, data-loading overhead was negligible relative to the computational cost of network training. The training time of 1 epoch in this setup was approximately 11 seconds on our dataset with a batch size of 256, with the 10-fold cross-validation results taking approximately 2 hours on the full dataset. The time required to train using this method will vary heavily depending on the hardware used and additional processes running during execution.

## Results

### Batch effect reduction through learnable features

We trained models using the adversarial learning method in each of the 10 stratified cross-validation folds. The resulting learned importance weights can then be used to transform the original spectra into a de-biased representation, which we feed into the final classification architecture.

**Fig. 6:**
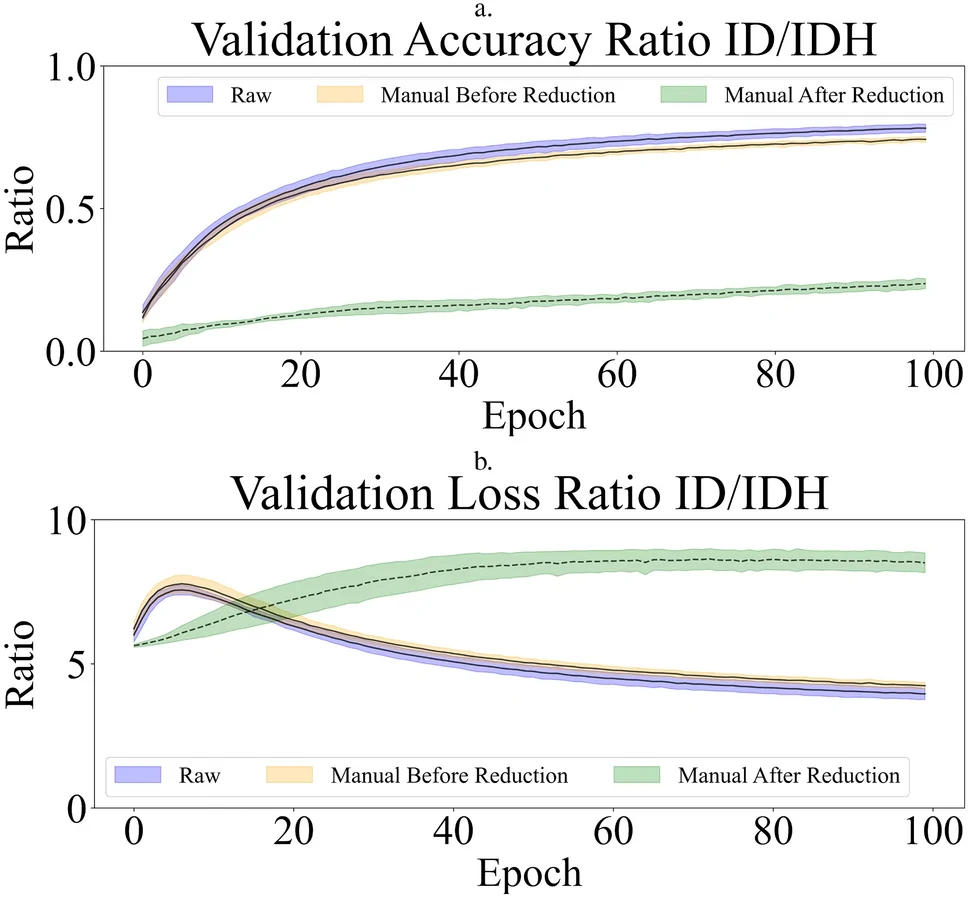
The ratio between ID and IDH1 metrics calculated as $\frac{{ID}}{{IDH1}}$. a. The validation accuracy and standard deviations across 10 folds of the raw- (blue), manually curated data before reducing the batch effect(orange), and manually curated data after reducing the batch effect(green). The accuracy increase between epochs is significantly smaller compared to the other datasets containing the batch effect. The same ratio is calculated using the validation loss metric. The loss is efficiently decreased for predicting ID in the raw and manually curated data while ID is harder to learn in the batch effect reduced data.

To evaluate the effect of the feature encoder we re-trained the architecture displayed in Fig. 2 using a learning rate of *1e-5* to stabilize the learning process. We monitored the increase in validation accuracies for both ID and IDH1 during training and noted that the model had a harder time increasing ID accuracy compared to that of IDH1. These results showed that the batch effect was drastically reduced, making it harder to model the relationships between spectra and their original sample IDs. The ratio between ID metrics and IDH1 metrics was calculated and compared across all training epochs to show the balance between batch effect and target learning. In Fig. 6, the ratios between ID accuracy and IDH1 accuracy (Fig. 6 a.), and ID loss and IDH1 loss (Fig. 6 b) are shown.

**Fig. 7:**
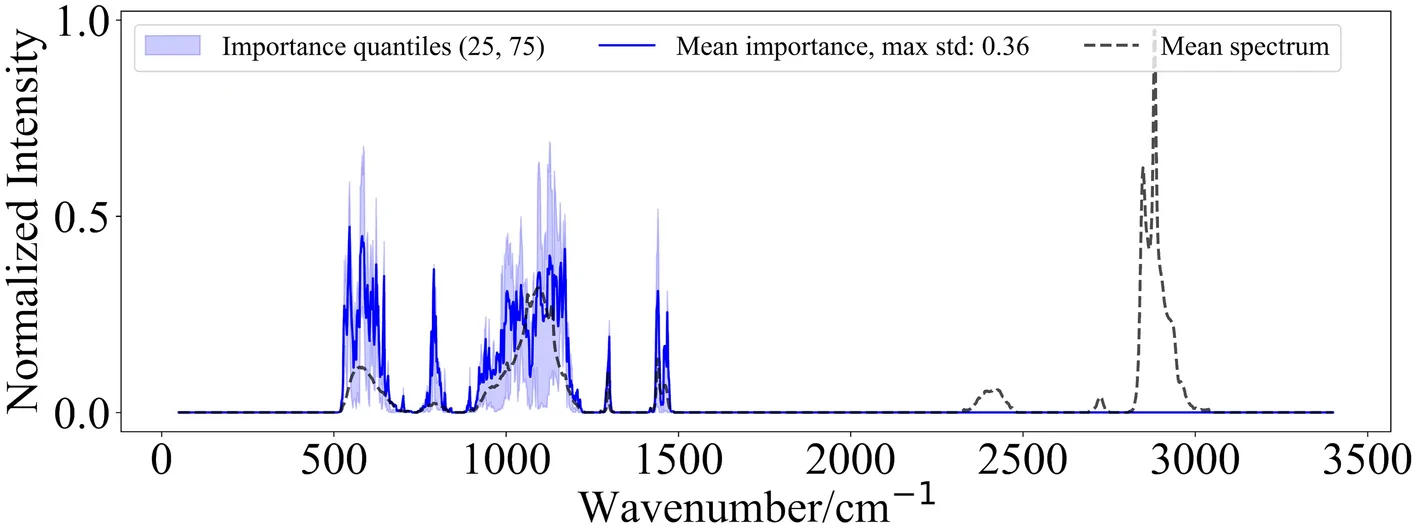
The feature importance vector learned through the suggested adversarial learning pipeline. In blue is the mean feature importance across all folds. The inter-quartile range between 25% to 75% of the 10 folds is shown in light blue. The black dotted spectrum is the mean spectrum of the available data. The maximum standard deviation among all learned feature vector wavelengths was calculated at 0.36.

**Fig. 8:**
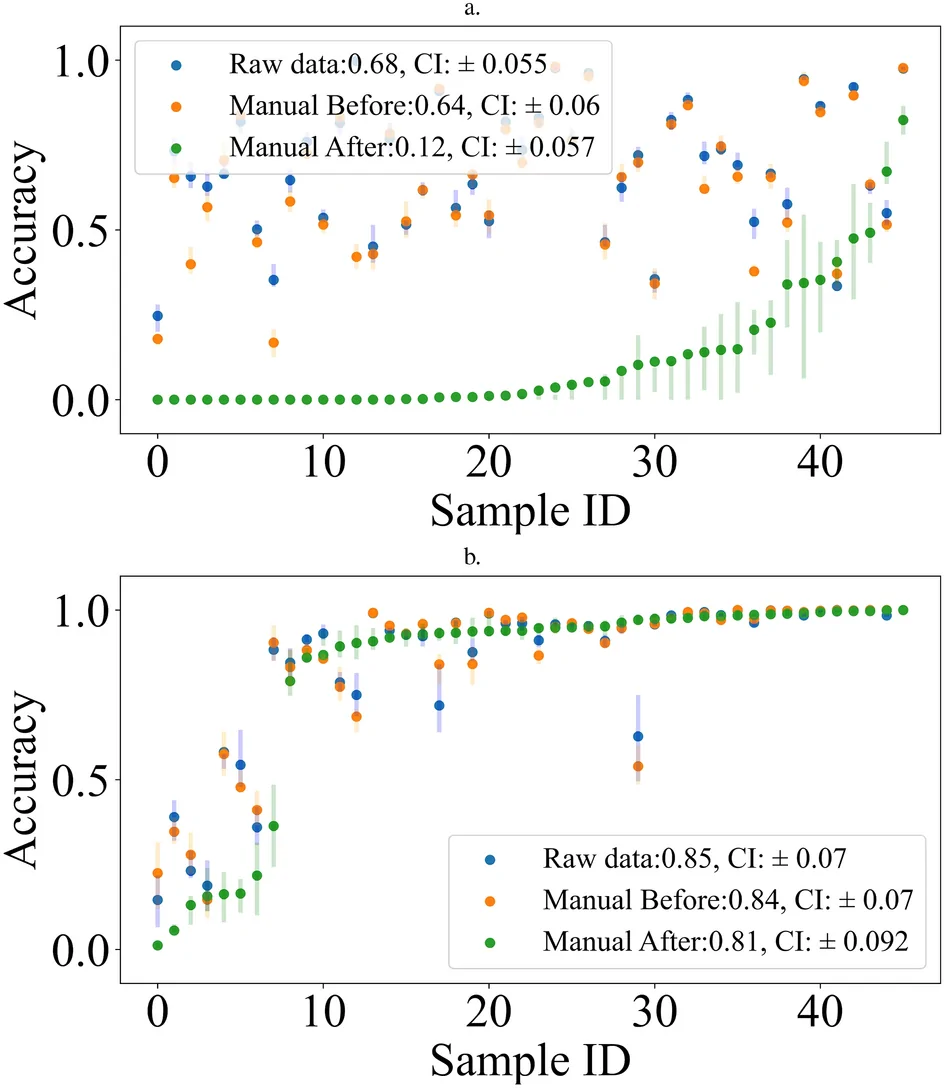
a. The validation accuracy of predicting the sample id on the raw data (blue), manually curated data (orange) and the manually curated data after removing the unimportant features (green). The dots represent the mean value of sample accuracies while the lines intersecting them represent the inter-quartile range (25, 75) across the 10 validation folds. The mean balanced validation accuracy was reduced from *68%* of the raw data to *12%* after applying our method, indicating a reduction in the batch effect. The horizontal axis indicates the sample IDs while the vertical indicates the accuracy on the sample. b. The mean balanced validation accuracy of predicting Mut vs. Wt per sample, on the same data as in b. The average balanced accuracy was reduced from *85%* on the raw data to *81%* by applying our method, indicating preservation of genetic information.

The mean learned feature importance’s following the application of our method on the available data is shown in Fig. 7. Following this process, the average balanced accuracy dropped to 12% for the sample ID task (Fig. 8 a), while remaining relatively high at 0.8 for the Mut vs. Wt classification (Fig. 8 b). These results indicate a successful mitigation of the batch effect: predicting the sample ID has becomes more difficult, while classifying the IDH1 mutation status remains competitive. This shows that some of the features useful for identifying IDH1 status were entangled with sample-specific signals. By selectively suppressing those entangled features, the model gets to focus on biologically relevant patterns alone.

Across all folds, the learned features are centered on the first half of the spectrum wavelengths. This suggested that the peaks present at the right half of the spectra could be ignored when learning IDH1 classification. By ignoring features whose computed importance are less than $\frac{1}{|N|}$ (where |*N*| is the number of features) in the mean feature importance vector (Fig. 7), the number of retained features is 392. The decreased batch effect in our data was determined to be substantial through analysis of Fig. 8.

Comparison between the validation accuracies on the batch effect reduced data in Fig. 8 a. between the manually preprocessed data and the resulting batch effect reduction showed a remarkable improvement. This suggests that our method could be applied to Raman datasets to significantly reduce the batch effect in datasets and lessening the risk of bias in analyses. Furthermore, The biological interpretation and relevance of the dataset was already discussed in our earlier publication^16^. For example, the peak at 532 cm-1 corresponds to cholesterol esters (such as cholesteryl stearate and palmitate), a novelty reported in our earlier publication to characterize IDH1mut against IDH1WT tumors was captured by the feature importance layer.

## Discussion

We demonstrated how the batch effect complicates the training of deep learning models and how it can be reduced via feature selection combined with adversarial training strategies. Raman spectra are information-rich and contain valuable information about biological samples; this information can be examined using the entire spectrum or by selecting a few wavelengths where documented molecular activity has been documented. Utilizing deep learning to analyze Raman spectra has tremendous promise since the feature learning capabilities of convolutional layers can be leveraged for feature engineering. The batch effect occurs in part due to problematic intensity regions which distract from the truly important regions and due to heterogeneous patterns which arise when comparing different wavelengths among different samples. With deep learning we can locate and remove non-trivial patterns to focus the training on the research question itself. We showed this through adversarial learning by training a feature importance layer to produce features which the connected deep learning model associated with the research question while removing features which distracted from it. Our results show the potential to limit the batch effect for Raman spectroscopy data and can be utilized to prepare novel Raman datasets for analysis and model training. Progress in this field is of vital importance as more data becomes available, enabling large-scale projects for spectra within combined cohorts. We view our work as a step in this direction, aiming to use computational methods to solve these issues and promote further research in this field.

We saw that spectra from one sample can be used to generalize to other unseen spectra in that same sample with relative ease. The use of baseline correction, cosmic ray removal, and denoising, which are standard methods for processing Raman spectra, can be used to marginally limit the batch effect, but does not remove it. Due to this, cross-validation remains the recommended method for developing classification methods for spectral sample datasets, since this removes the chance of generalizing on the spectra within samples by allocating them to the validation folds. The patterns which cause the batch effect can be non-trivial and appear to depend heavily on the experimental conditions, sample preparation, and equipment. These are factors which will remain variable in the foreseeable future, and so it falls to projects like ours to unify the samples for comparative analysis.

We show that the batch effect can be reduced through machine learning, by identifying and removing the features (Raman wavelengths) most responsible for the batch effect, while making sure that the research question being studied can still be answered with high accuracy with the remaining features. This implicitly indicates two aspects that can limit the applicability of our method. The first limiting aspect is that the Raman data contains a broad enough spectrum of wavelengths. This is important because our method identifies wavelengths to be removed from the entire dataset to reduce the batch effect, leaving potentially too few wavelengths to continue the project on. This can be the case for example when the data is coming from a stimulated Raman dataset, as opposed to the spontaneous Raman dataset we demonstrated our method on. The second limiting aspect is that there should be enough Raman spectra in the dataset to allow for an adversarial AI learning exercise. In our case we had available about 300.000 spectra on which we trained a model with thousands of parameters. Acquiring such a large dataset may not always be possible, as it implies the availability of a Raman mapping/imaging spectrometer and relevant materials.

The samples available in this project have a rich and heterogeneous makeup. In these analyses, it is common to have histological labels denoting the tumor type of each individual sample. Glioma tumors possess a strong heterogeneous makeup, which significantly complicates the classification of sample wide labels given individual spectra. We notice the heterogeneity in large part by predicting the sample ID; given sufficiently many epochs, even small models (number of parameters *< 10%* of the total dataset size) can achieve impressive accuracies on unseen data when using individual spectra from one sample to predict the sample ID of unseen spectra from that same sample in spite of inter-tumor heterogeneity. The combinations of features within the dataset can be utilized to predict almost any non-random label attached to the data. We measured the degree to which individual wavelengths influence this ability and discovered that any one feature is insufficient to properly predict the sample ID of the entire sample (maximum accuracy was < 8*%*). We concluded that at least 2 features are required for expressing the sample ID in the spectra. Our approach aimed to find and rank combinations of features which lacked sample ID expression but sufficiently expressed mutant vs. wild-type status of individual spectra. While our results did succeed in finding such features, the features also struggled to capture the IDH-status of the spectra, meaning it is possible the method removed too many features.

Possible improvements to our approach may lie in the encoding method where we utilized a customized feature importance layer. This layer develops a feature vector which is independent from the input spectrum. An improvement to this approach may be to utilize non-linear transformations of the input via convolutional and densely connected layers to encode the input into a batch effect reduced form. Since heterogeneity can introduce highly sparse variations between spectra, one could also create an encoder which aims to transform all spectra closer to the mean spectrum of a sample, thereby reducing the sample variances. Another possible improvement lies in the model architecture within which the batch effect is to be measured. In our experiments we utilized a model architecture which has a bottleneck before the output layer, i.e., the number of units in some latent layers was less than the number of output units. We believe this forced the model to reduce the batch effect naturally due to reduced latent size. Furthermore, we aimed to utilize small models in terms of the number of parameters; this may have unintentionally limited our experiment to simplistic data patterns rather than the possibly complex patterns spanning sample heterogeneity. One could increase the number of filters in the convolutional layers to increase the parameter count but we limited this to maintain computation and memory efficiency in our experiments.

The batch effect represents only one component of the broader reproducibility challenge in Raman spectroscopy and machine learning, including inter-patient biological variability, instrument-to-instrument differences, acquisition protocol variability, preprocessing choices, and limitations in model validation procedures. Recent discussions in the Raman spectroscopy literature^38^ have highlighted that methodological shortcomings in machine-learning evaluation can substantially inflate reported diagnostic performance. Therefore, reducing batch effects alone is not sufficient to ensure clinical translation; rigorous validation and reproducible evaluation protocols remain equally important.

Our method is mainly applicable in cases where the data has a hierarchical structure, being separable into different patient samples as in our examples. We designed it to address cases where the batch effect appears in non-trivial ways i.e. where the Raman frequencies are comparable between samples. This is in contrast to cases where the Raman peaks can have positional- and intensity variations. Significant variations between data cohorts would in this case require a pre-emptive unifying method of transformation, making the features comparable throughout the dataset. In this work, the batch effect presents itself as the minute differences in spectral patters which models can learn to utilize. These spectra appeared homogeneous across samples, but upon investigation were found to inhabit non-trivial and sample-specific patterns. From our results, it appears the method of determining feature importance with respect to the classification performance of the model on different tasks is a sufficient strategy to mitigate the batch effect in this context.

In comparison to other methods for batch effect mitigation (ComBat) and feature importance learning (LassoNet), our method is focused at performing both feature learning and batch effect mitigation at the same time. This way, we allocate the effort for batch effect removal to the feature importance layer while measuring the success in terms of the classification accuracies of the models. A suggestion for future studies in this field would be to combine ComBat and LassoNet to create a comparative solution to ours.

While the application could also be applied to datasets akin to the ones used by^34^ it has not been developed for or tested on those datasets explicitly. One potential future research project would be to apply our model to the datasets used by^34^ to establish the efficacy of our method on other datasets and to establish comparison between model approaches.

Our feature importance layer is designed to be used in the input layer, but it can be utilized at any 1-dimensional latent layer in the model architecture. In our experiments, the layer exhibited good separating capabilities for removing unimportant features while maintaining features relevant to the target variables. While the learnable parameters do express the importance for their relative features, the value of them may also be attributed to the bias within the model itself. We have observed cases where models start to overfit, using the noise present in unimportant features to increase training accuracy. In these cases, our feature importance layer denoted said features as important due to the model’s change in tactic, utilizing the available data distribution to increase predictive performance on seen data. Therefore, the feature importance layer can also be seen as a type of diagnostic layer, which not only can show the important features in datasets but also show how models utilize the data itself to make predictions. Further extensions of this method could be done by introducing heuristic limitations on the layer. For example, a minimum number of available data features could be imposed to prevent the layer from removing too many features and avoiding over-filtering. We note that these heuristics will be heavily reliant on the type of data available. Through examining the learnability of the data given singular features, we established that more than one feature was essential to map spectra to different target values. The use of a learnable feature layer through element-wise vector multiplications, enables the model to discard and rescale features as necessary.

Future prospects for these methods are aimed at enabling Raman spectroscopy at a larger scale and in real time within medical settings. For this reason we have focused our work on relatively shallow models compared to state-of-the-art DNNs. Practical application of these models inside clinical settings necessitate that the models can run on portable hardware and that their operations execute rapidly. By relying on small model architectures we seek to satisfy these requirements. Applying this method to novel, large datasets in a similar fashion to our case is going to be dependent on the number of samples in the dataset combined with the severity of the batch effect and the available hardware.

## Conclusion

Our approach managed to reduce the batch effect in a Raman dataset from 68% to 12%. It achieved this through the strategy outlined by Kheiri et al.^33^ which measures the batch effect by training a classifier neural network. The extent of the batch effect in a dataset is then defined as the accuracy of the final model. Our contribution combined this approach with a learnable feature importance layer, trained as an adversary to the classifier. Another novelty of our method is the use of binary mode outputs, which enabled the preservation of contextually relevant features. The final result of our approach is feature scaling for each feature, resulting in transformed data points with a reduced capability for informing patient IDs.

## Data acquisition

Unstained formalin-fixed paraffin-embedded (FFPE) tissue sections were analyzed as received, without further processing or modification. Spectra were collected at ambient laboratory temperature (22$^\circ$C–24$^\circ$C). Raman spectra were acquired on a ThermoFisher DXR2xi Raman microscope equipped with 532 nm single frequency laser diode, which was set to deliver 10 mW of power at the sample with a standard grating 50–3500 $\text {cm}^{-1}$ range, 4 $\text {cm}^{-1}$ resolution and a high sensitivity electron-multiplied CCD detector. The microscope was equipped with an Olympus air objective 10x/NA 0.25, MPlanN FN22. The scanning spectral range was 50 - 3400 $\text {cm}^{-1}$.

Data acquisition was performed using a 50 micron confocal pinhole, 2 s exposure time, 5 scans, image pixel size 5 mm, no photobleaching, EM gain on, and a medium baseline correction over the entire spectral window (50–3400 $\text {cm}^{-1}$) as previously described^16^.

The instrument performs an automatic calibration immediately after the laser is turned on. Instrument alignment was carried out periodically using a proprietary Thermo Fisher Scientific alignment tool, involving multi-source illumination through a pinhole and subsequent acquisition of polystyrene spectra to optimize laser alignment. These additional experimental details ensure reproducibility of the spectral acquisition protocol.

Some spectra may appear noisy due to the limited exposure time per pixel, which was intentionally chosen to prevent overheating and potential damage to the sample. Additionally, residual paraffin may contribute to the observed noise levels. The samples are inherently heterogeneous in chemical composition, and certain tissue regions may therefore exhibit higher noise than others.

In principle, there should be no peaks in the silent regions, but some signal is sometimes observed, due to interference of paraffin. As no background was removed, and because the spectra were min-max normalized, some humps might appear significant as shown in supplementary Fig. 9 a., and fluorescence can be visible in Fig. 3 a. This strengthens the necessity to address the batch effect especially on raw data. In the end, the majority of the frequencies, are positioned correctly as previously published frequency intervals. In our previous study^16^, during data processing, the silent region of the spectra was removed, and the remaining spectral segments were stitched together; thus, the final dataset did not include the silent region. In this study, the silent region was preserved in all of the three methods used to pre-process the data to not artificially eliminate any possible component of the batch effect.

## Supplementary Information

Supplementary Information.

## Data Availability

The code and data used in this project are available at https://github.com/JoelSjoberg/RamanSpectrumEffectGithub.
