# Supplementary material for: Deep learning mitigates batch effects in Raman spectroscopy
Source: Sci Rep. 2026 Oct 26;16:34019. doi: 10.1038/s41598-026-62086-1 (PMC13696425; doi:10.1038/s41598-026-62086-1)
Supplement: Supplementary file 1 [file 41598_2026_62086_MOESM1_ESM.pdf]

Supplementary Material

Table 1. The larger model trained for assessing the batch effect on the entire dataset. The diagram showing each layer with its corresponding parameters for the model trained to reduce the batch effect. Total number of parameters is 1815 of which 1773 are trainable.

Layer name	Parameters	# of parameters
Feature Encoder		
Flatten	-	0
FeatureImportance1D	inp shape = 1738	1740
Expand dimension	axis = -1	0
AvgeragePooling1D	pool size = 25, strides = 1	0
L2 Normalization	-	0
Conv1D	filters = 6, kernel size = 22, strides = 11	138
BatchNormalization	-	24
LeakyReLU	-	0
Conv1D	filters = 7, kernel size = 5, strides = 3	217
BatchNormalization	-	28
LeakyReLU	-	0
Conv1D	filters = 8, kernel size = 3, strides = 3	176
BatchNormalization	-	32
LeakyReLU	-	0
MaxPooling1D	pool size = 5, strides = 5	0
Flatten	-	0
L2 Normalization	-	0
ID prediction		
Flatten	-	0
Dense	size = 46	1150
Softmax	-	0
Mut vs. Wil prediction		
Flatten	-	0
Dense	size = 2	50
Softmax	-	0

Table 2. The smaller model for assessing the batch effect between two samples. The diagram showing each layer with its corresponding parameters for the small model trained to capture the batch effect between two samples. The model uses valid padding for convolutions. Total number of parameters is 665 of which 623 are trainable.

Layer name	Parameters	# of parameters
Conv1D	filters = 6, kernel size = 22, strides = 11	138
BatchNormalization	-	24
LeakyReLU	-	0
Conv1D	filters = 7, kernel size = 5, strides = 3	217
BatchNormalization	-	28
LeakyReLU	-	0
Conv1D	filters = 8, kernel size = 3, strides = 3	176
BatchNormalization	-	32
LeakyReLU	-	0
MaxPooling1D	pool size = 5, strides = 5	0
Flatten	-	0
L2 Normalization	-	0
ID prediction		
Flatten	-	0
Dense	size = 2	50
Softmax	-	0

Table 3. The sample-vise accuracy of sample ID and mutant vs. wildtype classification (IDH1)for the raw dataset. The number of spectra in each separated set is also listed to show the relative size between training and validation

	Training Accuracy			Validation Accuracy		
	sample ID	IDH1	Size	sample ID	IDH1	Size
0	0,35	0,95	6.461	0,36	0,95	718
1	0,96	0,15	2.995	0,96	0,15	333
2	0,82	1,00	6.501	0,82	1,00	722
3	0,51	0,96	3.653	0,52	0,96	406
4	0,83	0,99	2.423	0,82	0,99	269
5	0,54	1,00	5.621	0,54	1,00	625
6	0,97	1,00	12.926	0,98	1,00	1.436
7	0,92	0,99	6.108	0,92	0,99	679
8	0,45	0,96	3.627	0,45	0,96	403
9	1,00	1,00	5.200	1,00	0,99	578
10	0,94	0,88	3.203	0,94	0,88	356
11	0,53	0,95	9.030	0,52	0,95	1.003
12	0,55	0,39	9.621	0,55	0,39	1.069
13	0,57	0,93	4.406	0,57	0,93	490
14	0,46	0,79	3.542	0,46	0,79	394
15	0,66	0,99	3.231	0,65	0,99	359
16	0,35	0,94	3.624	0,35	0,94	403
17	0,64	0,91	3.842	0,64	0,91	427
18	0,71	0,23	3.459	0,72	0,23	384
19	0,84	0,73	1.870	0,82	0,72	208
20	0,66	0,98	3.673	0,66	0,98	408
21	0,52	0,96	3.532	0,53	0,96	392
22	0,25	0,96	3.500	0,25	0,96	389
23	0,74	0,59	3.043	0,74	0,58	338
24	0,34	0,92	6.776	0,34	0,91	753

Continued on the next page

Table 3 – continued from the previous page

	Training Accuracy			Validation Accuracy		
	sample id	IDH1	Size	sample id	IDH1	Size
25	0,63	0,97	3.276	0,63	0,96	364
26	0,66	0,98	3.605	0,66	0,99	401
27	0,83	1,00	6.574	0,82	1,00	730
28	0,88	0,75	3.600	0,88	0,75	400
29	0,63	0,96	16.708	0,63	0,96	1.856
30	0,76	1,00	3.298	0,76	1,00	366
31	0,76	0,88	3.093	0,76	0,88	344
32	0,73	0,62	3.362	0,73	0,63	374
33	0,63	1,00	5.492	0,62	1,00	610
34	0,68	1,00	10.810	0,69	1,00	1.201
35	0,83	0,55	3.628	0,83	0,54	403
36	0,50	0,96	1.840	0,50	0,95	204
37	0,72	0,36	3.026	0,72	0,36	336
38	0,91	0,92	2.991	0,91	0,92	332
39	0,63	0,17	2.958	0,62	0,19	329
40	0,67	0,84	4.630	0,67	0,84	514
41	0,98	0,99	4.149	0,98	0,98	461
42	0,57	0,90	7.672	0,58	0,91	852
43	0,77	0,98	6.474	0,77	0,98	719
44	0,74	1,00	7.102	0,74	1,00	789
45	0,86	0,93	8.673	0,86	0,93	964

Table 4. The sample-vise accuracy of sample ID and mutant vs. wildtype classification (IDH1) for the manually processed dataset. The number of spectra in each separated set is also listed to show the relative size between training and validation

	Training Accuracy			Validation Accuracy		
	sample ID	IDH1	Size	sample ID	IDH1	Size
0	0,34	0,96	6.461	0,34	0,96	718
1	0,95	0,22	2.995	0,95	0,22	333
2	0,80	1,00	6.501	0,80	1,00	722
3	0,53	0,97	3.653	0,52	0,97	406
4	0,84	0,99	2.423	0,83	0,99	269
5	0,52	1,00	5.621	0,52	1,00	625
6	0,98	1,00	12.926	0,98	1,00	1.436
7	0,90	0,99	6.108	0,90	0,99	679
8	0,44	0,96	3.627	0,43	0,96	403
9	0,42	0,99	5.200	0,42	0,99	578
10	0,94	0,90	3.203	0,94	0,90	356
11	0,38	0,94	9.030	0,38	0,95	1.003
12	0,52	0,35	9.621	0,52	0,35	1.069
13	0,54	0,94	4.406	0,54	0,93	490
14	0,46	0,78	3.542	0,46	0,77	394
15	0,59	1,00	3.231	0,58	0,99	359
16	0,17	0,95	3.624	0,17	0,95	403
17	0,66	0,87	3.842	0,66	0,87	427
18	0,64	0,27	3.459	0,62	0,28	384
19	0,85	0,82	1.870	0,84	0,84	208
20	0,70	0,99	3.673	0,70	0,99	408
21	0,55	0,98	3.532	0,54	0,98	392

Continued on the next page

Table 4 – continued from the previous page

	Training Accuracy			Validation Accuracy		
	sample ID	IDH1	Size	sample ID	IDH1	Size
22	0,18	0,95	3.500	0,18	0,95	389
23	0,75	0,58	3.043	0,75	0,58	338
24	0,37	0,90	6.776	0,37	0,90	753
25	0,58	0,96	3.276	0,57	0,96	364
26	0,40	0,97	3.605	0,40	0,97	401
27	0,82	1,00	6.574	0,81	1,00	730
28	0,87	0,68	3.600	0,87	0,69	400
29	0,64	0,97	16.708	0,63	0,97	1.856
30	0,74	1,00	3.298	0,72	1,00	366
31	0,76	0,84	3.093	0,76	0,84	344
32	0,66	0,55	3.362	0,65	0,54	374
33	0,62	1,00	5.492	0,62	1,00	610
34	0,66	1,00	10.810	0,66	1,00	1.201
35	0,82	0,47	3.628	0,82	0,48	403
36	0,48	0,95	1.840	0,46	0,94	204
37	0,69	0,42	3.026	0,70	0,41	336
38	0,91	0,96	2.991	0,92	0,96	332
39	0,66	0,15	2.958	0,66	0,15	329
40	0,65	0,84	4.630	0,66	0,83	514
41	0,98	1,00	4.149	0,98	1,00	461
42	0,53	0,89	7.672	0,52	0,88	852
43	0,79	0,98	6.474	0,78	0,98	719
44	0,70	1,00	7.102	0,70	1,00	789
45	0,85	0,86	8.673	0,85	0,86	964

Table 5. The sample-vise accuracy of sample ID and mutant vs. wildtype classification (IDH1) for the RADAR processed dataset. The number of spectra in each separated set is also listed to show the relative size between training and validation

	Training Accuracy			Validation Accuracy		
	sample ID	IDH1	Size	sample ID	IDH1	Size
0	0,35	0,97	6.461	0,36	0,97	718
1	0,95	0,10	2.995	0,95	0,10	333
2	0,78	1,00	6.501	0,78	1,00	722
3	0,52	0,97	3.653	0,52	0,97	406
4	0,85	0,99	2.423	0,85	0,99	269
5	0,52	1,00	5.621	0,52	1,00	625
6	0,96	1,00	12.926	0,96	1,00	1.436
7	0,93	1,00	6.108	0,93	0,99	679
8	0,41	0,97	3.627	0,41	0,97	403
9	0,91	0,98	5.200	0,91	0,99	578
10	0,91	0,83	3.203	0,90	0,83	356
11	0,37	0,92	9.030	0,37	0,92	1.003
12	0,49	0,37	9.621	0,50	0,37	1.069
13	0,52	0,94	4.406	0,52	0,94	490
14	0,47	0,82	3.542	0,47	0,81	394
15	0,55	0,99	3.231	0,54	0,99	359
16	0,23	0,96	3.624	0,24	0,96	403
17	0,68	0,86	3.842	0,68	0,86	427
18	0,65	0,27	3.459	0,64	0,27	384

Continued on the next page

Table 5 – continued from the previous page

	Training Accuracy			Validation Accuracy		
	sample ID	IDH1	Size	sample ID	IDH1	Size
19	0,87	0,87	1.870	0,87	0,88	208
20	0,67	0,98	3.673	0,67	0,98	408
21	0,53	0,98	3.532	0,53	0,98	392
22	0,25	0,95	3.500	0,25	0,96	389
23	0,74	0,58	3.043	0,74	0,58	338
24	0,35	0,89	6.776	0,35	0,89	753
25	0,57	0,95	3.276	0,59	0,95	364
26	0,61	0,98	3.605	0,61	0,98	401
27	0,84	1,00	6.574	0,84	1,00	730
28	0,86	0,82	3.600	0,86	0,81	400
29	0,62	0,97	16.708	0,62	0,97	1.856
30	0,76	1,00	3.298	0,75	1,00	366
31	0,75	0,81	3.093	0,75	0,80	344
32	0,70	0,60	3.362	0,70	0,61	374
33	0,62	1,00	5.492	0,61	1,00	610
34	0,63	1,00	10.810	0,63	1,00	1.201
35	0,78	0,55	3.628	0,78	0,55	403
36	0,54	0,97	1.840	0,53	0,98	204
37	0,68	0,39	3.026	0,67	0,40	336
38	0,90	0,97	2.991	0,90	0,97	332
39	0,63	0,15	2.958	0,64	0,15	329
40	0,63	0,85	4.630	0,63	0,85	514
41	0,97	0,99	4.149	0,97	0,99	461
42	0,62	0,90	7.672	0,62	0,90	852
43	0,78	0,99	6.474	0,78	0,99	719
44	0,71	1,00	7.102	0,71	1,00	789
45	0,87	0,93	8.673	0,87	0,93	964

Algorithm 1 Batch effect reduction

Require: $E \geq 1$
  $e \leftarrow 0$
  Perform spectral curation
    Baseline removal
    Cosmic ray removal
    Denoising
  Outlier detection
    Compute quantile between 0.005 and 0.995 for every wavenumber.
    Locate spectra containing intensities outside the quantile range for any wavenumber
    Remove the located spectra.
  Identify dataset enumeration characteristic of the batch effect b.
  while $e < E$ **do**
    Lock the feature encoding layer
    Train the model to increase accuracy on t and b
    Lock the model parameters and unlock the feature importance parameters
    Train the model to minimize b accuracy and increase t accuracy.
    $e \leftarrow e + 1$
  end while
  Return learned feature encoder

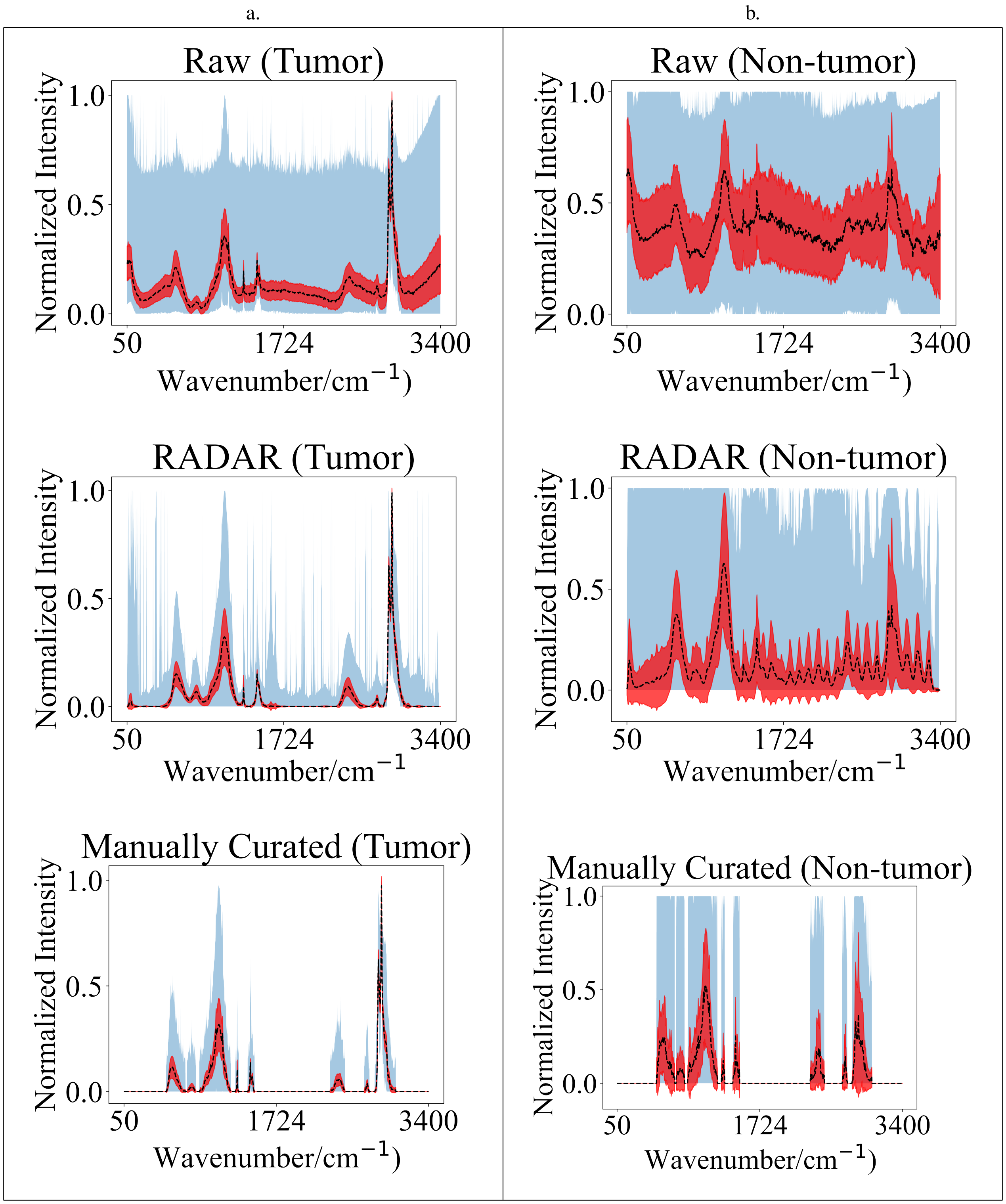

Figure 9. a. The distribution of the tumor spectra within the dataset. The first row shows the tumor spectra from the raw data, the second row shows the RADAR corrected data, and the third row shows the manually curated data. The black dotted line is the mean of the spectra, the red area shows the standard deviation from the mean and the blue background area shows the span between minimum to maximum intensities for all spectral features. b. The outliers identified within the corresponding data versions.

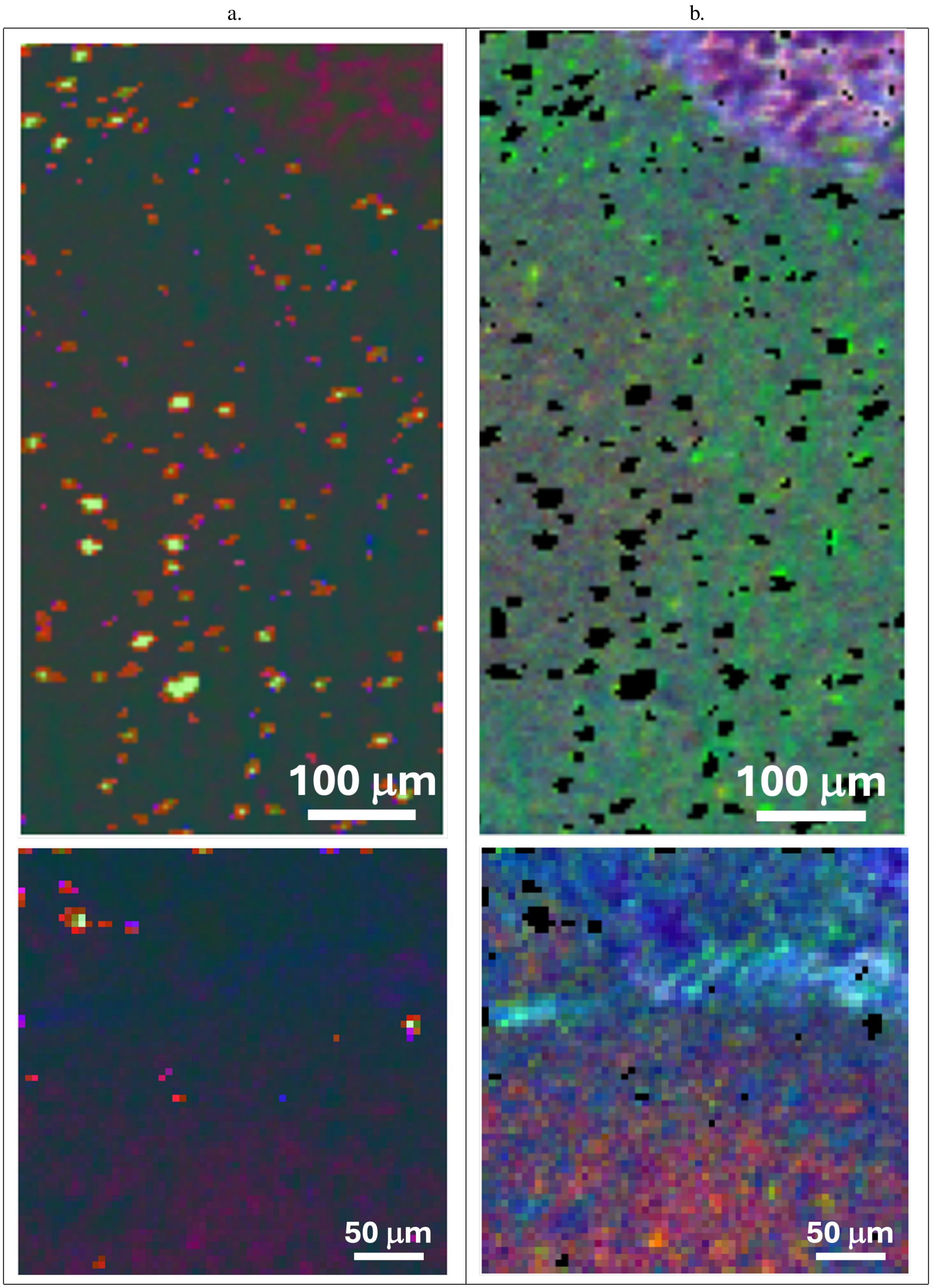

Figure 10. a. The tumor surfaces of two distinct samples before outlier removal utilizing PCA (3 components) to produce a 2-dimensional surface; the outliers differ from the tumor spectra to the extent where the diversity of the surface cannot be properly inspected. b. The heterogeneous makeup is clearly shown after outlier removal. The black areas show locations of outlier spectra while colored areas show the tumor surface.

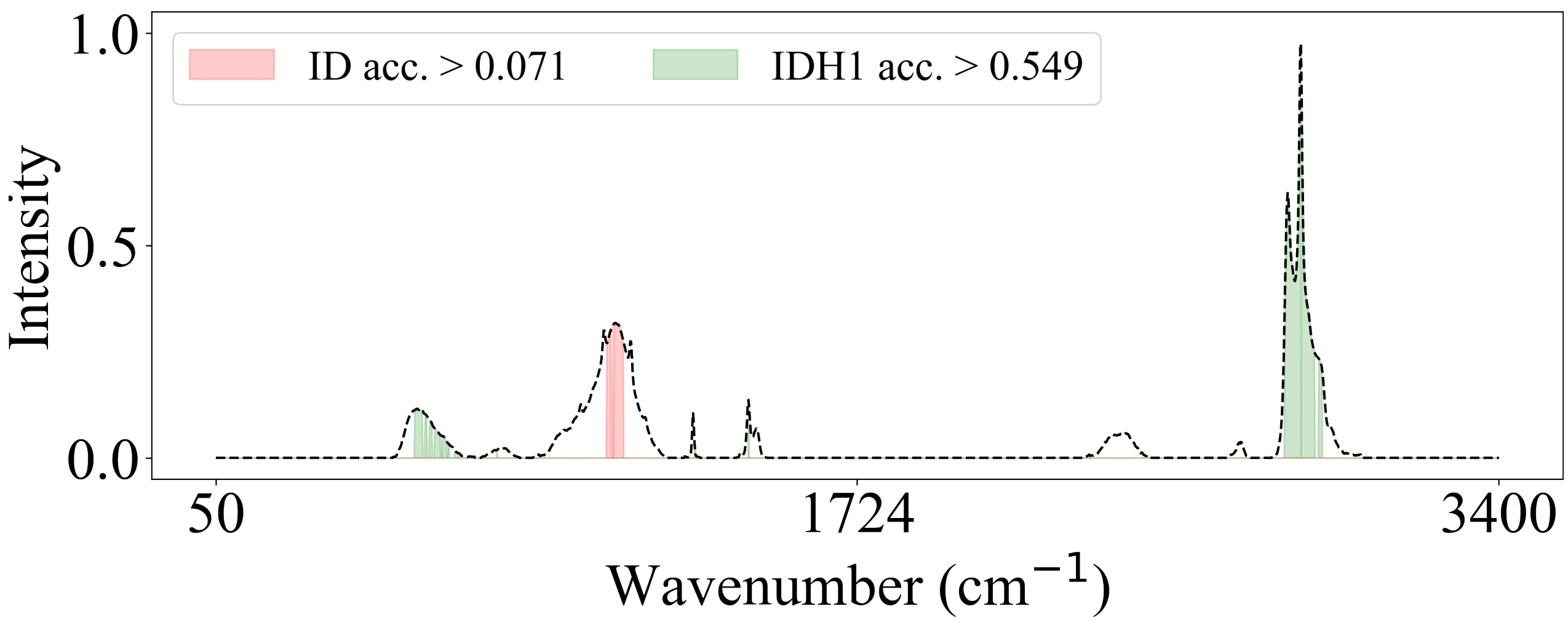

Figure 11. Assessing the contribution of individual Raman frequencies to the batch effect. We display the mean spectrum over the entire training dataset. The frequencies consisting of 0-intensities are removed to magnify the Raman peaks. No single feature alone is sufficient to accurately predict the sample ID and the IDH1 mutation status. However, some individual features did achieve balanced accuracy over the baseline prediction for sample ID classification (higher than the frequency of the most frequent category 7.1%) and IDH1 status (higher than the frequency of the majority class 55%). The results displayed are based on the mean accuracies of the 10 validation folds. In red: the frequencies which for which the decision trees achieved an average accuracy higher than the baseline for ID classifications. In light green: the frequencies which the decision trees achieved an average accuracy higher than the baseline for IDH1 classifications.
